# P-1299. Clinical Outcomes of Ertapenem in Critically Ill Patients with Bacteremia Caused by ESBL-Producing Organisms: A Comparison of Normal/elevated and Low Albumin Levels

**DOI:** 10.1093/ofid/ofaf695.1487

**Published:** 2026-01-11

**Authors:** Brian Chung, Barbara Kamel, Kirby An, Henry Donaghy, Juby Roy, Reshma George, Aya Haghamad, Thien-Ly Doan

**Affiliations:** Long Island Jewish Medical Center, Flushing, NY; Long Island Jewish Medical Center, Flushing, NY; North Shore University Hospital, Oakland Gardens, NY; Donald and Barbara Zucker School of Medicine at Hofstra/Northwell, Dayton and Karen Brown Division of Infectious Diseases, New Hyde Park, New York; Donald and Barbara Zucker School of Medicine at Hofstra/Northwell, Dayton and Karen Brown Division of Infectious Diseases, New Hyde Park, New York; Donald and Barbara Zucker School of Medicine at Hofstra/Northwell, Dayton and Karen Brown Division of Infectious Diseases, New Hyde Park, New York; Northwell, Lake Success, New York; Long Island Jewish Medical Center, Flushing, NY

## Abstract

**Background:**

Ertapenem, a highly protein-bound drug, may lead to an increased unbound fraction of drug in those with hypoalbuminemia, resulting in faster metabolism and excretion. Because critically ill patients commonly experience hypoalbuminemia, the IDSA guidance suggests utilizing meropenem over ertapenem. This study evaluated whether serum albumin levels impact clinical outcomes the critically ill treated with ertapenem for bacteremia caused by extended-spectrum beta-lactamase (ESBL) producing organisms.

**Figure:**
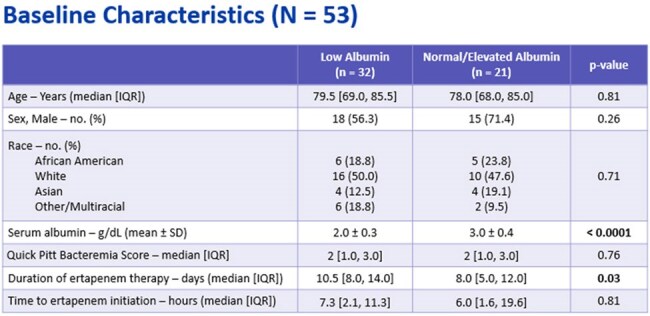
Baseline Characteristics The study groups were well matched between the low and normal/elevated albumin groups (e.g., age, sex, similar severity of bacteremia, and time to initiation of ertapenem.

**Figure:**
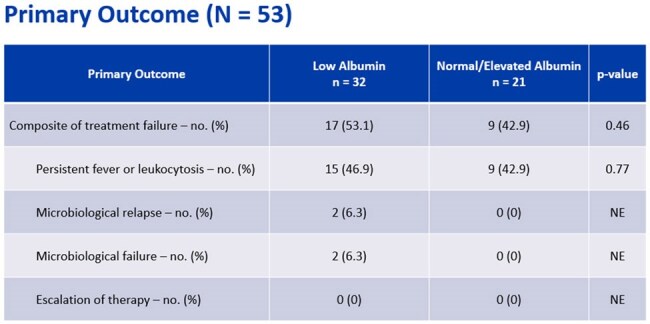
Primary Outcomes Patients receiving ertapenem in the low albumin group had more treatment failure than normal/albumin, although this was not statistically significant. The numbers were driven by microbiologic failure and relapse.

**Methods:**

This IRB-exempt, multi-center, retrospective review of ICU patients with bacteremia with polymerase chain reaction (PCR) detection of CTX-M gene from 2021-2024. Patients were included if ertapenem was initiated within 48 hours of PCR detection and continued for ≥ 2 days. Exclusion criteria included ertapenem resistance, > 48 hours of another carbapenem prior to ertapenem, or discharge/death within 48 hours. Patients were stratified into low albumin and normal/elevated albumin (e.g., < 2.5 g/dL and ≥ 2.5 g/dL). Data collected included demographics, antibiotic use, and laboratory values. The primary outcome was a composite endpoint of treatment failure defined as persistent fever or leukocytosis, microbiological failure/relapse, or escalation of therapy. Secondary outcomes included length of stay (LOS), time to microbiological eradication, and 30-day all-cause in-hospital mortality. Appropriate statistical analysis was performed.

**Figure:**
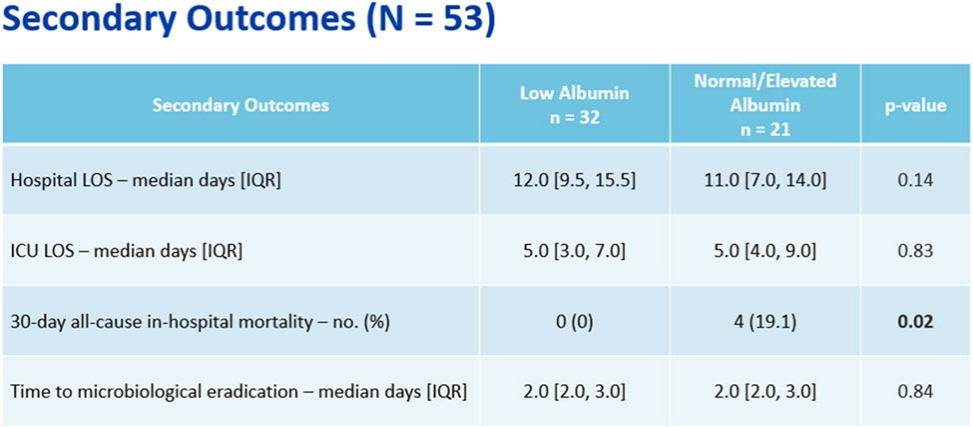
Secondary Outcomes There was no difference in hospital and ICU length of stay in patients with low versus normal/elevated albumin. There was no difference seen in time to microbiological eradication. The 30-day all-cause mortality was higher in then normal/elevated albumin (n = 4 versus none in the low albumin group), but all but 1 patient cleared their bacteremia and had resolution of leukocytosis and fever.

**Figure:**
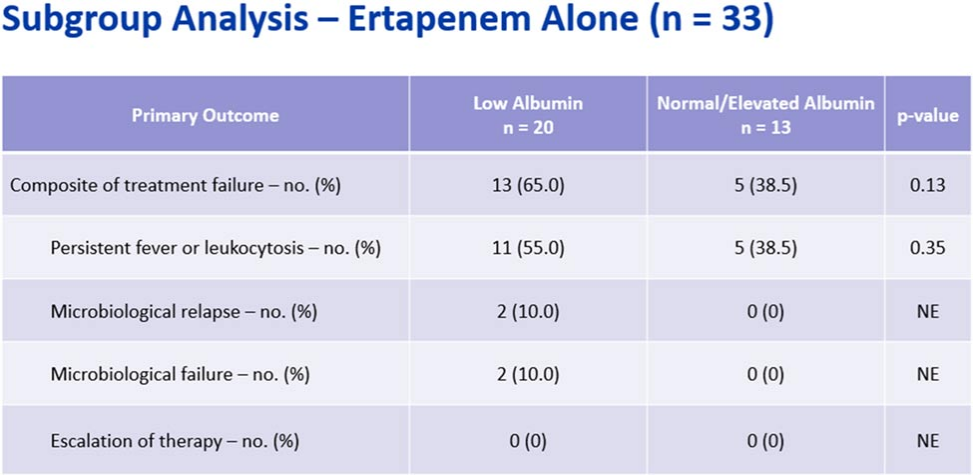
Subgroup Analysis - Ertapenem Alone Although not statistically significant, there were more treatment failures in the low albumin group, including 2 patient with microbiological relapse and failure.

**Results:**

A total of 53 patients were included: 32 in low albumin and 21 in the normal/elevated group. Treatment failure occurred more frequently in the low albumin (53.1% vs. 42.9%, p = 0.46). No differences were seen in hospital or ICU LOS, or time to microbiological eradication. In those receiving ertapenem monotherapy, treatment failure was higher in the low albumin group (65% vs. 38.5%, p = 0.13). When meropenem was used as lead-in therapy, treatment failure was not seen. Study limitations include a small sample size and use of adjunctive therapy.

**Conclusion:**

Among critically ill patients with ESBL bacteremia treated with ertapenem, those with low albumin had numerically higher treatment failure. Ertapenem may remain a viable option in those with hypoalbuminemia if they receive meropenem as lead-in therapy, prior to de-escalation.

**Disclosures:**

All Authors: No reported disclosures

